# Amino Acid Restriction Impairs Human Endometrial Stromal Cell Decidualization and Is Rescued by Proline Supplementation

**DOI:** 10.1111/jcmm.70821

**Published:** 2025-09-16

**Authors:** Chloe Jang, Cristiana Iosef, Stephen J. Renaud, Victor K. M. Han

**Affiliations:** ^1^ Department of Biochemistry Western University London Canada; ^2^ Department of Anatomy and Cell Biology Western University London Canada; ^3^ Children's Health Research Institute London Canada; ^4^ Department of Pediatrics Western University London Canada

**Keywords:** decidua, fetal growth restriction, growth factors, halofuginone, leucine, placenta, preeclampsia, proline

## Abstract

Decidualization is a critical process for successful pregnancy. It is characterised by the transformation of endometrial stromal cells into decidual cells that support embryo implantation and placental development. Maternal amino acid deficiency is linked to impaired decidualization, which can lead to pregnancy complications such as miscarriage, preeclampsia and fetal growth restriction. Halofuginone (HF), a synthetic alkaloid, induces nutritional stress by triggering the amino acid starvation response. This study investigated the effects of HF‐induced nutritional stress and amino acid supplementation on decidualization of human endometrial stromal cells (HESCs). Exposure of HESCs to decidualization agents caused distinct morphological changes and expression of insulin‐like growth factor binding protein‐1 (IGFBP‐1), indicative of successful decidualization. Treatment of HESCs with HF or leucine‐deprived media inhibited the expression of key decidualization markers. Interestingly, supplementation with proline, but not leucine, rescued the inhibitory effects of HF on decidualization of HESCs. HF inhibited the expression of genes encoding growth factors crucial for decidualization, highlighting their sensitivity to amino acid availability, and disrupted transforming growth factor β‐SMAD signalling, which was restored by proline supplementation. These findings highlight the essential role of amino acids, particularly proline, for proper decidualization and suggest potential therapeutic strategies for improved reproductive health.

## 1 Introduction

Maternal nutrition plays a critical role in ensuring a successful pregnancy and optimal fetal growth and development [1]. Inadequate protein intake restricts amino acid availability, impairing placental function and nutrient transport to the fetus with lasting consequences for the offspring [2]. The decidua is a specialised uterine mucosal layer derived from the endometrium that forms the base of the placental bed. Decidualization, a dynamic process initiated during the luteal/secretory phase of the menstrual cycle and augmented if fertilisation occurs, is essential for implantation and maintaining a healthy pregnancy. Defects in this process are implicated in recurrent spontaneous abortion [3], fetal growth restriction [4] and preeclampsia [5]. Previous studies highlight the importance of amino acid availability for human decidual cell function, notably regarding branched‐chain amino acids and L‐tryptophan [6, 7], and glutamic acid for decidualization in mice [3]. However, the impact of amino acid restriction on the decidualization process is not well understood.

Decidualization in humans is initiated in response to changes in cyclic adenosine monophosphate (cAMP) and progesterone‐driven steroid hormone signalling. During this process, human endometrial stromal cells (HESCs) undergo a morphological shift from fibroblast‐like cells to polygonal epithelioid cells [8]. This is associated with dynamic changes in the expression of genes associated with epithelial to mesenchymal transition (EMT) [9], as well as signalling through growth factors. For instance, vascular endothelial growth factors (VEGF) and platelet‐derived growth factors (PDGF) promote decidual cell motility [10], and proangiogenic factors such as VEGF, hepatocyte growth factor (HGF) [11], fibroblast growth factors (FGF) [12] and angiopoietins (ANGPT) [12] support endovascular remodelling. Decidual stromal cells are also the primary source of maternal insulin‐like growth factor‐binding protein‐1 (IGFBP‐1) during pregnancy in most mammals, including humans [13]. IGFBP‐1 is a hallmark of HESC decidualization and plays a key role in embryo implantation and cellular growth by sequestering IGFs, thereby preventing their interaction with receptors and reducing their growth‐promoting activity [14]. Prolactin (PRL) is also produced by decidual stromal cells to support placentation, maternal adaptations and immunomodulation [15]. Transcription factors heart and neural crest derivatives expressed 2 (HAND2) and forkhead box O1 (FOXO‐1) are upregulated during decidualization and regulate *IGFBP‐1* and *PRL* expression [8].

The transforming growth factor β (TGFβ) superfamily, including TGFβ1‐3, activin, nodal and bone morphogenetic proteins (BMPs), regulates decidualisation by influencing endometrial cell growth, adhesion, migration and cell fate (reviewed in [16]). Binding of TGFβs or BMPs to their cognate receptors leads to phosphorylation of SMAD2/3 (for TGFβ) or SMAD1/5/8 (for BMP), which then complex with SMAD4 to regulate gene expression. In *Smad3*‐null mice, decidualisation is severely compromised, and genetic depletion of *Smad2* and *Smad3* in mouse decidual cells reduces the expression of decidual prolactin‐related protein, which is a decidualisation marker in mice [17]. In humans, the role of SMAD2 and SMAD3 in decidualisation is not well understood.

Amino acid restriction triggers stress responses to adapt to nutrient scarcity. Since TGFβ‐SMAD signalling has nutrient‐sensing roles [18] and is crucial for decidualisation, we hypothesised that amino acid restriction influences HESC differentiation through this pathway. We used Halofuginone (HF), an alkaloid from *Dichroa febrifuga*, as a well‐recognised agent to simulate amino acid restriction and induce an intracellular stress response [19]. HF inhibits prolyl‐tRNA synthetase, causing uncharged tRNA accumulation and mimicking amino acid scarcity [20]. We found that HF impaired decidualisation, which was restored by proline but not leucine supplementation. HF inhibited TGFβ‐SMAD signalling, which was also rescued by proline. These findings highlight the importance of amino acid sensing pathways in decidual formation, offering insights into pregnancy complications linked to maternal protein restriction.

## 2 Materials and Methods

### 2.1 In Vitro Decidualization of HESCs

Immortalised HESCs from Applied Biological Materials (T0533, Richmond, BC, Canada) or American Type Culture Collection (ATCC, CRL‐4003, Manassas, VA, USA) were maintained in phenol red‐free DMEM/F12 (1:1) media (21041‐025, Gibco, Billings, MT, USA) with 10% fetal bovine serum (FBS; Gibco), 100 U/mL penicillin and 100 μg/mL streptomycin (Gibco). Cells were passaged before confluence using 0.05% Trypsin–EDTA (Gibco). For decidualisation, cells were seeded at 4 × 10^4^ cells/mL in a 6‐well plate, and media were replaced the next day with control or decidualisation media. Control medium consisted of phenol red‐free DMEM/F12 with 2% dextran‐coated charcoal‐stripped FBS, 100 U/mL penicillin and 100 μg/mL streptomycin. To prepare decidualisation medium, 1 μM medroxyprogesterone (MPA; Cayman Chemicals, Ann Arbour, MI, USA) and 0.5 mM 8‐Br‐cAMP (Cayman Chemicals) were added to control medium. Media were replenished every 48 h. Cells were cultured in a humidified incubator with 5% CO₂ at 37°C.

### 2.2 Cell Treatments and Viability

HESCs were treated with HF (50 or 100 nM, 13370, Cayman Chemicals) for up to 8 days in decidualization medium. To assess amino acid supplementation, 2 mM proline (Cayman Chemicals) or leucine (Sigma‐Aldrich) was added, with or without HF. For leucine deprivation experiments, HESCs were decidualized for 6 days in leucine‐replete (450 μM) or leucine‐depleted (0 μM) medium. This medium was prepared from DMEM/F‐12 (D9785, Sigma‐Aldrich, Oakville, ON, Canada) by supplementing the missing amino acids to match standard DMEM/F‐12 composition, except for leucine, which was either added at 450 μM (equivalent to standard DMEM/F‐12) or omitted entirely (0 μM). Viability was measured by 3‐(4,5‐dimethylthiazol‐2‐yl)‐2,5‐diphenyltetrazolium bromide (MTT) assay (Sigma‐Aldrich).

### 2.3 Immunofluorescence

HESCs were seeded at 2 × 10^4^ cells/mL in chamber slides precoated with 0.2% (w/v) gelatin in Dulbecco's PBS (dPBS). After 6 days of decidualisation, cells were fixed with cold acetone. Then, fixed cells were rehydrated with dPBS and permeabilised with 0.02% Triton X‐100. After blocking with fish serum, primary antibodies (mouse IGFBP‐1, rabbit Vimentin, rabbit SMAD4; details in Table S1) were incubated overnight at 4°C. The next day, species‐appropriate, fluorophore‐conjugated secondary antibodies were applied, followed by staining with DyLight 554 Phalloidin and Hoechst 33342, and mounted with Fluoromount‐G (Thermo Fisher Scientific, Waltham, MA, USA).

### 2.4 Luciferase Assays

TGFβ‐SMAD signalling was assessed by transfecting HEK293T cells (CRL‐3216, ATCC) with a plasmid encoding nano‐luciferase downstream of a minimal promoter containing SMAD‐binding elements (SBE; CS177101, pNL[NlucP/SBE/Hygro], Promega, Madison, United States). HEK293T cells were seeded at 4 × 10^5^ cells/mL, transfected with ViaFect (Promega) for 24 h, and then treated for 2 h with 100 nM HF, 2 mM proline, 2 mM leucine and 50 ng/mL TGFβ1 (ab50038, Abcam, Cambridge, UK) alone or in various combinations. Luciferase activity was measured using the Nano‐Glo Luciferase Assay System (Promega) on a Victor3 microplate reader (PerkinElmer, Waltham, MA, USA).

### 2.5 Quantitative Reverse Transcriptase PCR (qRT‐PCR)

RNA was extracted using TRIzol (Thermo Fisher). RNA (500 ng) was reverse‐transcribed using the High‐Capacity cDNA Reverse Transcription kit (Thermo Fisher). The cDNA was diluted 1:10 and amplified with SensiFAST SYBR Lo‐ROX PCR Master Mix (FroggaBio, Toronto, ON, Canada) and primers (Table S2) on a CFX Connect Real‐Time PCR system (Bio‐Rad, Mississauga, ON, Canada). Cycling conditions included 95°C for 10 min, followed by 40 cycles of 95°C for 15 s and 60°C for 1 min, with a dissociation phase. Relative mRNA expression was calculated using the ΔΔCt method with *YWHAZ*, *ACTB*, and *GAPDH* as reference RNAs.

### 2.6 Enzyme‐Linked Immunosorbent Assay (ELISA)

IGFBP‐1 levels in culture media were quantified using an ELISA kit (DGB100, Biotechne R&D Systems, Minneapolis, MN, USA) with a sensitivity of 13.8 pg/mL. The kit detects free IGFBP‐1 but not IGFBP‐1 bound to IGF‐1 or IGF‐2. IGFBP‐1 concentrations were interpolated from a standard curve using recombinant IGFBP‐1.

### 2.7 Western Blotting

Conditioned media were collected, mixed with sample buffer (30% glycerol, 0.5 M Tris–HCl pH 6.8, 12% SDS), and heated at 90°C for 8 min. Equal protein amounts were loaded onto SDS‐polyacrylamide gels. Separated proteins were transferred to nitrocellulose membranes. Membranes were blocked with 5% skim milk and incubated overnight with primary antibodies (Table S1). IGFBP‐1 was detected using mouse monoclonal IGFBP‐1 6303 SP‐5, while phosphorylated IGFBP‐1 (Ser101) was detected using a custom antibody described previously [21]. Following incubation with peroxidase‐labelled secondary antibodies, bands were visualised using Clarity Max Western ECL substrate and imaged with the VersaDoc system (Bio‐Rad, Mississauga, ON, Canada). Densitometry was performed using Image Lab software (Bio‐Rad). Intracellular protein was collected in cell lysis buffer (Cell Signalling Technology, Danvers, MA, USA) containing protease and phosphatase inhibitors (Sigma‐Aldrich) and then quantified using the Bradford assay (Bio‐Rad) using OD 595 nm absorbance compared to a standard curve generated using known concentrations of bovine serum albumin. All values were normalised to the control, which was assigned a value of 1.0.

### 2.8 RNA Sequencing and Analysis

RNA was purified using TRIzol, followed by 70% ethanol dilution and purification on RNeasy columns with DNase I treatment to remove residual DNA contamination (Qiagen, Toronto, ON, Canada). RNA integrity was determined using an Agilent Bioanalyzer (Agilent Technologies, Santa Clara, CA, USA). All samples had an RNA integrity number > 9. Libraries were prepared using the Illumina Stranded mRNA Prep Kit. Sequencing was performed on an Illumina NovaSeq S4 platform Xp protocol with 100‐bp paired‐end reads, targeting ~25 M reads per sample (Illumina, San Diego, CA, USA). Data were analysed using Partek Flow (Illumina) and aligned to the human genome (GRCh38) using STAR 2.7.8a and annotated using hg38 Ensembl transcripts release 110. Features with fewer than 8 reads were filtered out. Normalisation was done by counts per million (cpm) plus 1.0. Differentially expressed genes (DEGs) were identified with an adjusted *p*‐value < 0.05 and Log2FC ≥ 1. Pathway analysis was performed using iPathway Guide, which retrieves from Kyoto Encyclopedia of Genes and Genomes (KEGG), Gene Ontology and BioGRID databases. Hallmark gene sets were visualised with SRplot [22] and TGFβ signalling genes (has04350) were analysed using Morpheus and STRING.

### 2.9 Data Presentation and Statistical Analysis

For RNA sequencing, statistical analysis was described above. For other experiments, comparisons between two groups were made using Student's *t*‐test, while comparisons of three or more groups were assessed using one‐way analysis of variance (ANOVA) with Tukey's post hoc test. A *p* value < 0.05 was considered statistically significant. GraphPad Prism 6.0 was used for graphing and statistical analyses, and each experiment was repeated at least three times.

## 3 Results

### 3.1 Validation of HESC Decidualization

**FIGURE 1:**
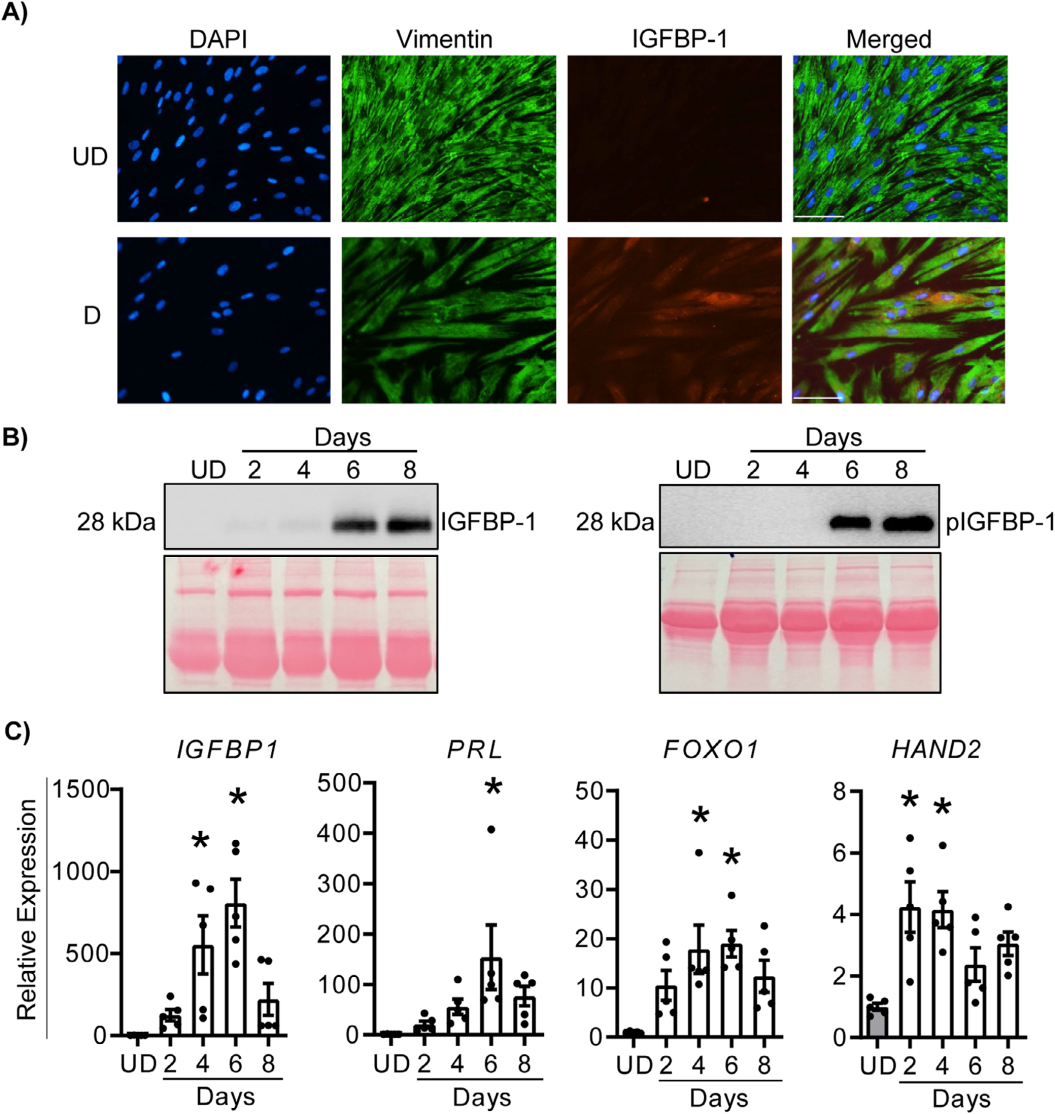
Decidualisation of HESCs. (A) Immunofluorescence for Vimentin and IGFBP‐1 in HESCs cultured in the presence of 0.5 mM 8‐Br‐cAMP and 1 μM MPA (decidualization media), (D) for 6 days. HESCs cultured without 8‐Br‐cAMP and MPA (undecidualized, UD) were used as controls. Nuclei were counterstained using DAPI. The scale bar represents 100 μm. (B) Western blot showing total and phosphorylated (Ser101) IGFBP‐1 in media conditioned by HESCs cultured in UD media or decidualisation media for up to 8 days. Ponceau stains show equal loading of the gels. (C) Transcript levels of *IGFBP1*, *PRL*, *FOXO1* and *HAND2* in HESCs cultured in UD or D media for up to 8 days. Data are presented as mean ± SEM. Asterisks represent statistical significance (*; *p* < 0.05) versus UD based on one‐way ANOVA with Tukey's multiple comparisons test. *N* = 3 for panels (A) and (B). *N* = 5 independent samples for panel (C). HESCs, human endometrial stromal cells.

To confirm that HESCs decidualised in the presence of MPA and 8‐Br‐cAMP, immunofluorescence for Vimentin, an intermediate filament protein, and IGFBP‐1 was conducted on HESCs following 6 days culture in control or decidualisation medium (Figure 1A). Cells cultured in decidualisation medium exhibited a more condensed epithelioid shape, unlike the elongated fibroblastic morphology of undecidualised HESCs. When cultured in decidualisation medium, levels of IGFBP‐1 were increased and fewer cells were observed, which is consistent with reduced cell cycle progression during differentiation (Figure 1A). Using western blotting, increased IGFBP‐1 and phosphorylated IGFBP‐1 (Ser101) were detectable in conditioned media from HESCs cultured in decidualisation medium, with peak levels on days 6 and 8 (Figure 1B). Furthermore, the expressions of *IGFBP‐1*, *PRL*, *FOXO1* and *HAND2* were increased in cells cultured in decidualisation medium (Figure 1C), confirming robust decidualisation of HESCs upon exposure to MPA and 8‐Br‐cAMP.

### 3.2 HF Inhibits Decidualization of HESCs

**FIGURE 2:**
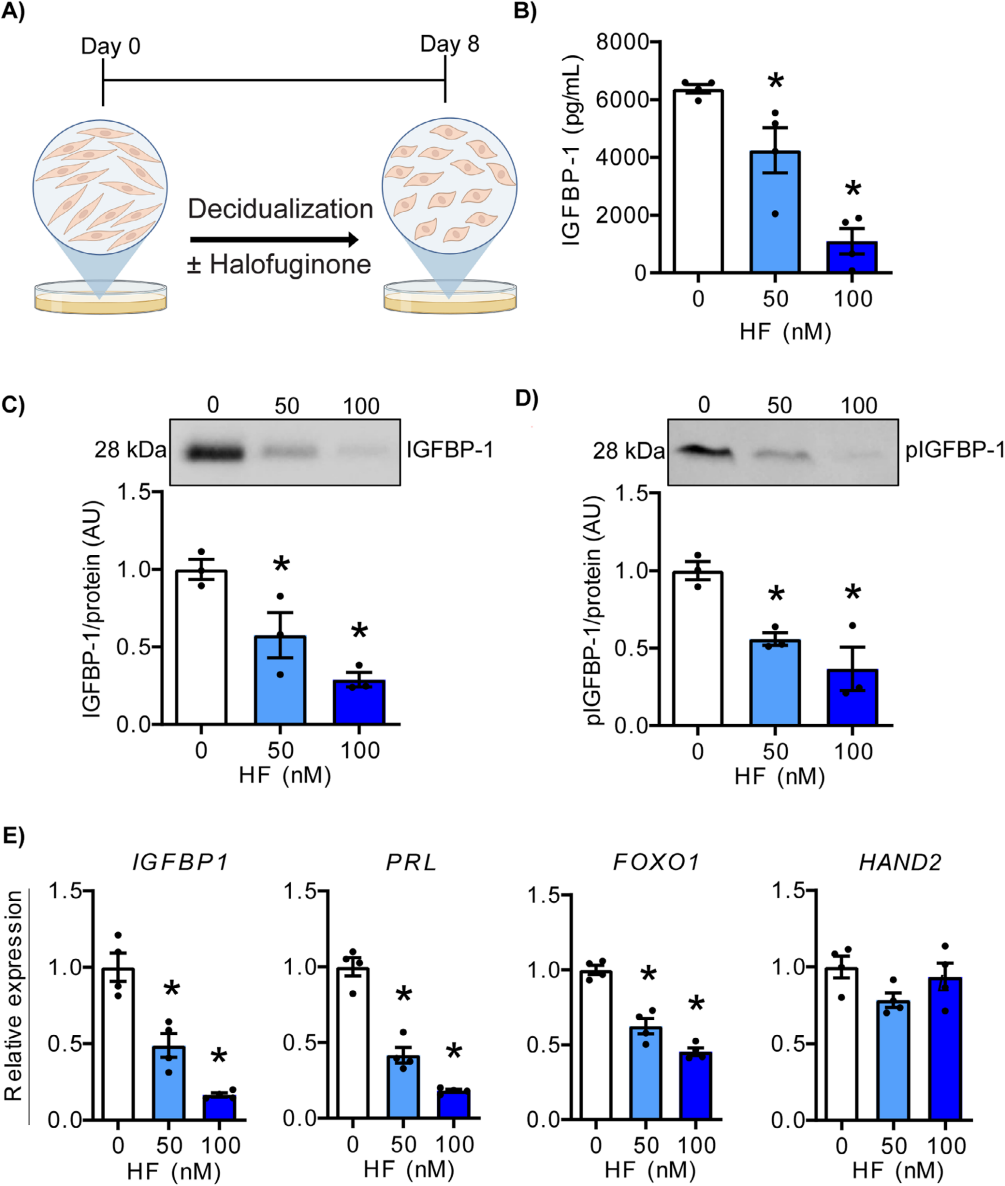
HF inhibits IGFBP‐1 secretion and decidualisation in HESCs. (A) Schematic of experimental design. HESCs were treated with decidualising agents for 8 days in the presence or absence of HF at doses of 50 or 100 nM. Created in BioRender (https://BioRender.com/4c2ulat). (B) Quantification of secreted IGFBP‐1 was performed using ELISA. (C, D) Representative western blots of (C) IGFBP‐1 and (D) phosphorylated (Ser101) IGFBP‐1 in media conditioned by HESCs cultured in the presence or absence of HF. (E) Relative transcript levels of *IGFBP1*, *PRL*, *FOXO1*, and *HAND2* in HESCs cultured in the presence or absence of HF. Data are presented as mean ± SEM. Asterisks represent statistical significance (*; *p* < 0.05) versus controls (0 HF) based on one‐way ANOVA with Tukey's multiple comparisons test. *N* = 3 independent replicates for panels (B)–(D), *N* = 4 independent replicates for panel (E). HESCs, human endometrial stromal cells; HF, halofuginone.

To determine whether activating the amino acid starvation response modulates decidualisation of HESCs, cells were cultured in decidualisation medium and treated with 50 or 100 nM HF for 8 days (Figure 2A). IGFBP‐1 and phosphorylated IGFBP‐1 levels in conditioned media decreased in a dose‐dependent manner, as measured by ELISA (Figure 2B; *p* < 0.05) and western blotting (Figure 2C,D; *p* < 0.05). Furthermore, the expressions of *IGFBP1*, *PRL* and *FOXO1* were significantly reduced in HESCs treated with HF (*p* < 0.05), while *HAND2* expression was unaffected (Figure 2E). These results indicate that HF likely impairs decidualisation through mechanisms independent or downstream of *HAND2*.

### 3.3 Proline but Not Leucine Supplementation Rescues Decidualisation of HESCs in the Presence of HF

**FIGURE 3:**
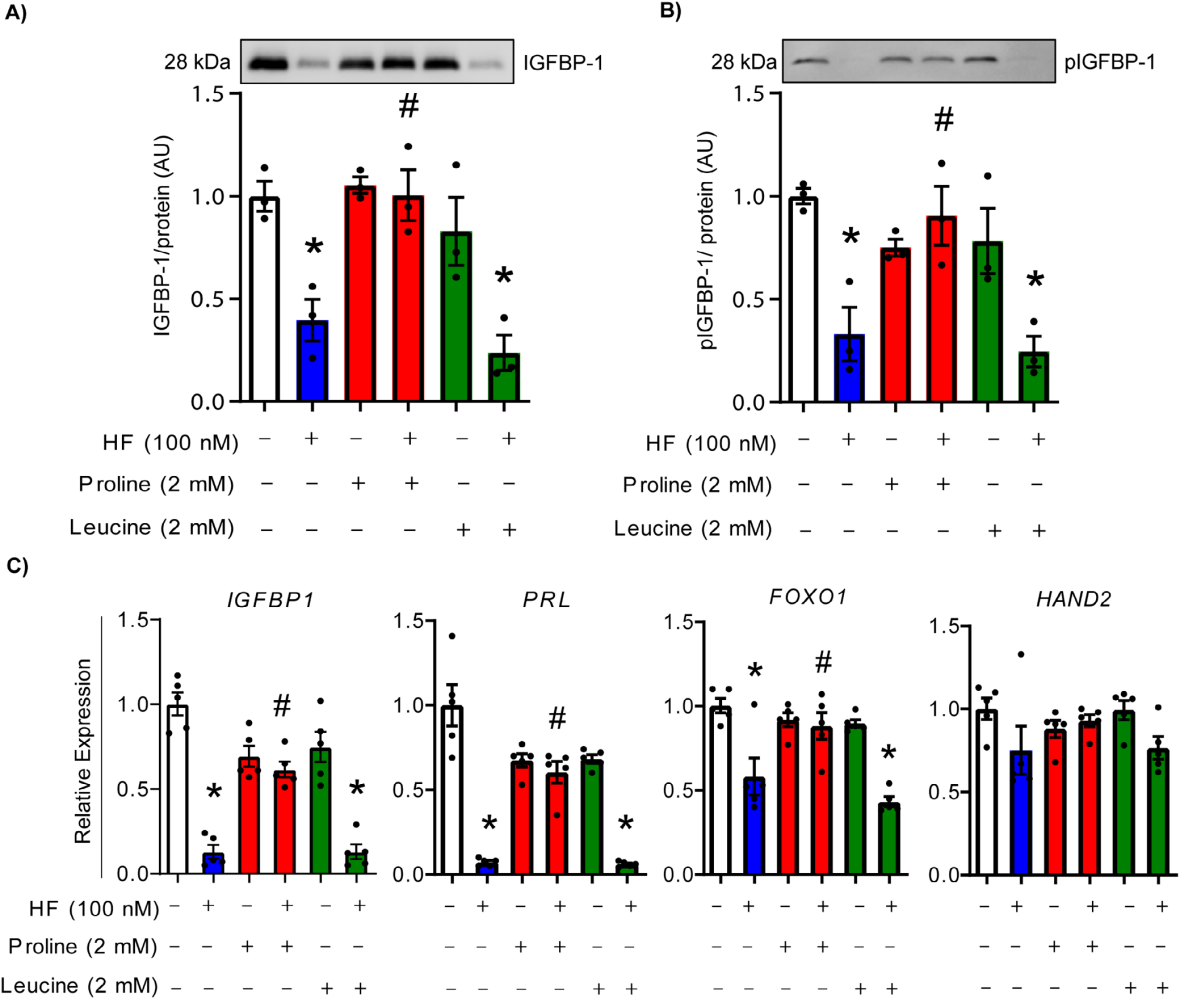
Proline supplementation rescues IGFBP‐1 secretion and decidualisation following HF treatment. HESCs were cultured in decidualisation media and treated with 100 nM HF for 8 days in the presence or absence of 2 mM Proline or Leucine. (A, B) Representative western blots are shown for levels of (A) IGFBP‐1 and (B) phosphorylated (Ser101) IGFBP‐1 in conditioned media. (C) Quantitative RT‐PCR was used to assess the expression of *IGFBP1*, *PRL*, *FOXO1* and *HAND2*. Data are presented as means ± SEM. Asterisks represent statistical significance versus untreated cells (*; *p* < 0.05), and number signs represent statistical significance when comparing HF‐treated cells to those supplemented with Proline or Leucine (#; *p* < 0.05), based on one‐way ANOVA with Tukey's multiple comparisons test. *N* = 3 independent replicates for panels (A) and (B), *N* = 5 independent replicates for panel (C). HESCs, human endometrial stromal cells; HF, halofuginone.

As HF acts by competing with proline for binding to glutamyl‐prolyl‐tRNA synthetase and blocking prolyl‐tRNA charging, we next determined whether the decidualization of HESCs could be rescued by proline supplementation to outcompete HF for binding to tRNA synthetase. Supplementation of cells with the neutral amino acid leucine was used as a negative control. HESCs were cultured in decidualization medium and treated with or without HF (100 nM) for 8 days, in the presence of either 2 mM proline or 2 mM leucine. In comparison to HF treatment alone, HESCs cultured in decidualization medium and treated with both HF and proline had increased levels of total IGFBP‐1 and phosphorylated IGFBP‐1 (both *p* < 0.05) in conditioned media, with levels comparable to that of HESCs not exposed to HF. This rescue effect was not observed when HESCs were treated with HF supplemented with leucine (Figure 3A,B). Notably, supplementation with proline or leucine in the absence of HF had no effect on levels of IGFBP‐1 or phosphorylated IGFBP‐1, and there were no changes in the number of viable cells between different treatment conditions (Figure S1). Supplementation with proline, but not leucine, restored the expression of *IGFBP1*, *PRL* and *FOXO1* in HF‐treated cells to levels comparable with controls (Figure 3C, all *p* < 0.05).

To determine whether the inhibitory effect on decidualization is specific to proline deficiency or applies more broadly to amino acid insufficiency, we examined the effects of leucine deprivation on HESC decidualization. HESCs were cultured in leucine‐replete (450 μM) or leucine‐depleted (0 μM) decidualization medium for 6 days. Similar to HF treatment, leucine deprivation significantly impaired decidualization, reducing IGFBP‐1 secretion and *IGFBP1*, *PRL* and *FOXO1* expression (Figure S2). These findings suggest that while HF exerts its effects through proline‐specific mechanisms, decidualization requires a broad availability of amino acids and is generally inhibited by the deficiency of certain amino acids such as leucine.

### 3.4 HF Inhibits Decidualisation and Influences Pathways Regulating Decidual Cell Function and Response to Stress

**FIGURE 4:**
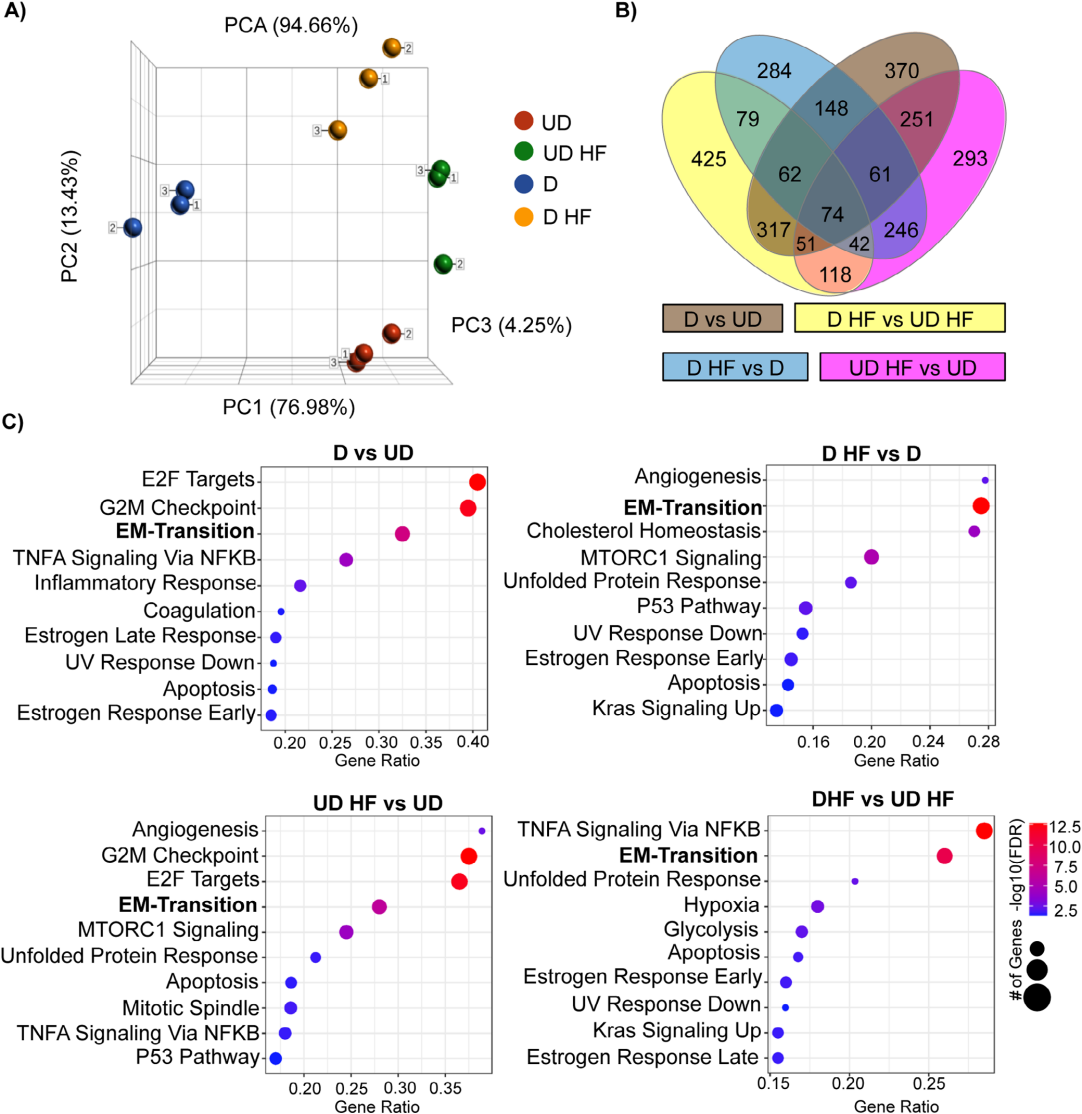
Whole transcriptome analysis reveals that HF inhibits decidualisation and affects pathways that regulate decidual cell function and stress responses. (A) Principal component analysis (PCA) illustrates the variability in the gene expression profiles of Undecidualised HESCs (UD), UD treated with HF (UD HF), Decidualizing HESCs (D) and D treated with HF (D HF). (B) Venn diagram is annotated with the number of DEGs between different contrasts. (C) Hallmark gene set analyses performed on iPathway Guide reveal the top 10 annotated pathways for each experimental contrast, where epithelial to mesenchymal (EM) transition appeared in all four contrasts. HESCs, human endometrial stromal cells; HF, halofuginone.

To gain mechanistic insight into how HF disrupts the early stages of HESC decidualization, we performed RNA sequencing on cells cultured in control or decidualization medium for 4 days with or without HF. The gene expression profiles of HF‐treated cells (in both control and decidualization medium) closely resembled those of undecidualized cells. In contrast, cells in decidualization medium without HF showed distinct profiles (Figure 4A). The number of DEGs between groups is presented in Figure 4B. Pathway analysis highlighted an enrichment of processes involved in cell cycle regulation, inflammatory responses and oestrogen signalling in cells undergoing decidualization. Pathways enriched in both decidualized and undecidualized cells treated with HF included angiogenesis, mechanistic target of rapamycin complex 1 (mTORC1) signalling, and the unfolded protein response. Interestingly, no pathways associated with the integrated stress response were annotated despite the well‐established connection of HF to this pathway (Figure 4C).

### 3.5 HF Disrupts the Expression of Genes Encoding Growth Factors Associated With Decidualisation

**FIGURE 5:**
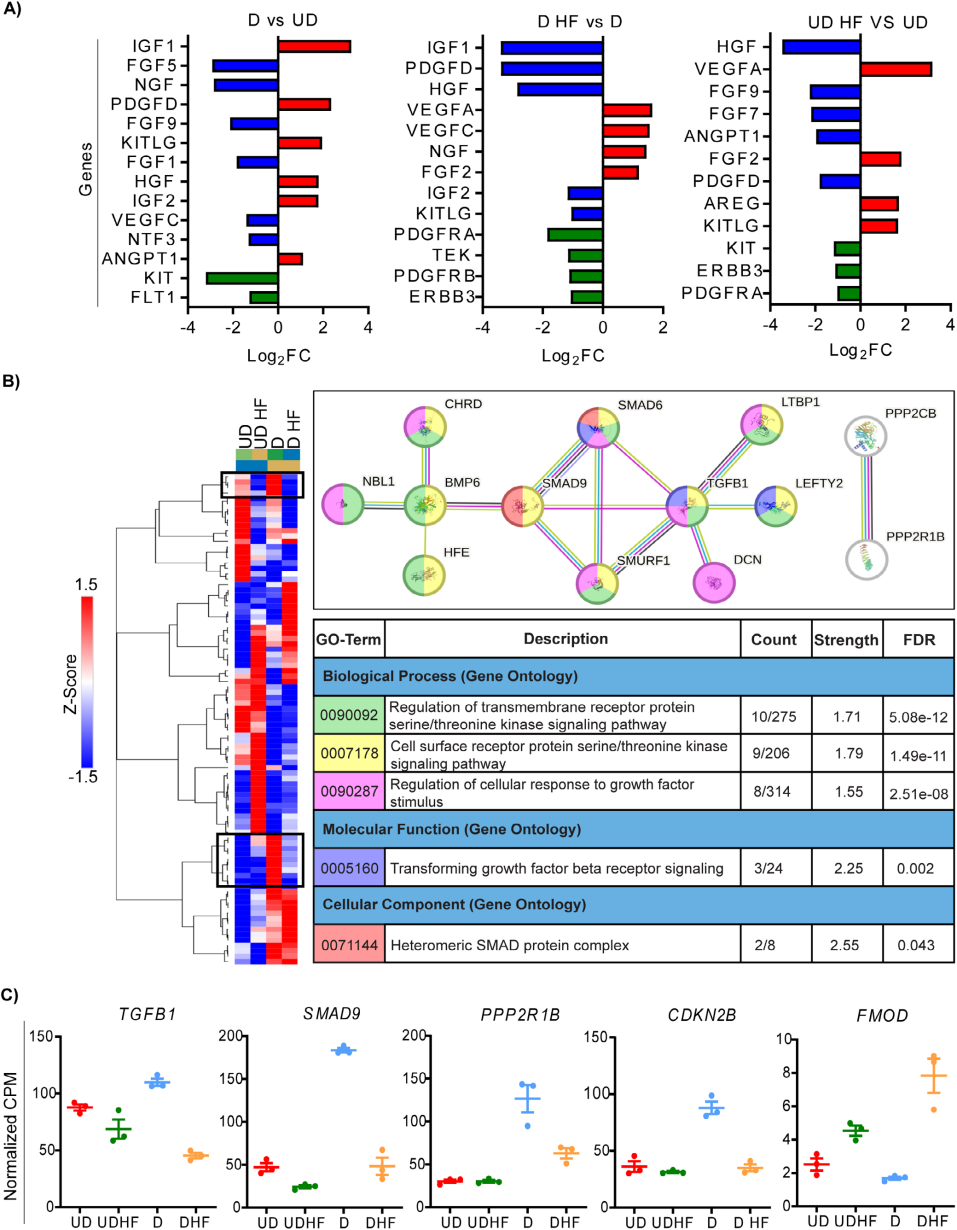
Decidualization of HESCs alters the expression of genes involved in growth factor signalling, which is impaired by HF. HESCs were treated with decidualization agents in the presence or absence of 100 nM HF. After 4 days, RNA was extracted and utilised for next generation sequencing. (A) The top differentially expressed genes encoding growth factors and receptor tyrosine kinases are shown for three contrasts: HESCs cultured in decidualizing media compared to undecidualizing media (D versus UD), HESCs cultured in decidualizing media with and without HF (D HF versus D) and HESCs cultured in undecidualizing media with and without HF (UD HF versus UD). Red and blue bars indicate genes encoding growth factors that were upregulated and downregulated, respectively. Green bars indicate receptor tyrosine kinases that were differentially expressed. (B) A heatmap showing gene expression values for the TGFβ signalling pathway was plotted for the four treatments. The expression values were normalised to counts per million (CPM) and then adjusted to reflect the *Z*‐score. Genes that increased expression with decidualization and decreased with HF (indicated in the black box) were plotted in STRING, with key GO terms indicated. (C) Normalised CPM of selected genes corresponding to the TGFβ signalling pathway: *TGFB1*, *SMAD9*, *PPP2R1B*, *CDKN2B* and *FMOD*. HESCs, human endometrial stromal cells; HF, halofuginone.

RNA sequencing revealed significant changes in the expression of genes encoding various growth factors between HF‐treated and untreated cells. In decidualization media, genes promoting growth (*IGF1*, *IGF2*, *HGF*), blood vessel maturation (*ANGPT1*, *PDGFD*) and cell survival (KIT ligand, *KITLG*) were upregulated, aligning with known decidualization‐associated transformations [23] (Figure 5A). HF treatment inhibited *IGF1*, *IGF2*, *PDGFD*, *HGF* and *KITLG* expression and increased the expression of neurotrophins like nerve growth factor (*NGF*) and angiogenic markers (*VEGFA*, *VEGFC*). Interestingly, the expression of growth factor‐encoding genes most affected by HF differed between cells cultured in control versus decidualization media, indicating that the effect of HF on growth factor signalling likely differs based on cell type and stage of differentiation (Figure 5A).

### 3.6 TGFβ‐SMAD Signalling Is Attenuated by HF in Decidualizing HESCs

When contrasting the expression profiles of HESCs in control or decidualization medium with or without HF, EMT emerged as the only hallmark gene set shared across multiple pairwise comparisons (Figure 4C). As components of the TGFβ signalling pathway are part of the EMT gene set and crucial for decidualization, we examined their expression profiles. In HESCs cultured in decidualization media, genes associated with canonical TGFβ‐SMAD signalling were significantly upregulated, including *SMAD9*, *SMAD6*, Inhibitor of Differentiation (*ID)‐1*, *ID3* and *ID4* (Figure S3A). Key regulators of cell growth and division were also upregulated, including *TGFB1*, *CDKN2B* and *PPP2R1B*. However, the expression of these genes was not increased in cells cultured in decidualization medium with HF, suggesting that HF interferes with this signalling trajectory (Figure S3B).

We next plotted the normalised cpm values of genes from the TGFβ signalling pathway (hsa04350; Figure 5B). Of particular interest was the subset of genes that were stimulated during decidualization but repressed by HF. We created a subnetwork of these genes using STRING (Figure 5B), where 13 members had at least one connection. These genes were associated with cellular responses to growth factors and receptor signalling regulation (Figure 5B). Key genes like *TGFB1*, *SMAD9*, *PPP2R1B* and *CDKN2B* had reduced expression under HF treatment, potentially due to increased *FMOD* expression, a TGFβ inhibitor (Figure 5C, Figure S3B). Taken together, our findings suggest that numerous genes associated with TGFβ signalling are dynamically regulated during decidualization, and this process is disrupted in cells treated with HF.

### 3.7 Proline Supplementation Mitigates the Impact of HF on TGFβ‐SMAD Signalling

**FIGURE 6:**
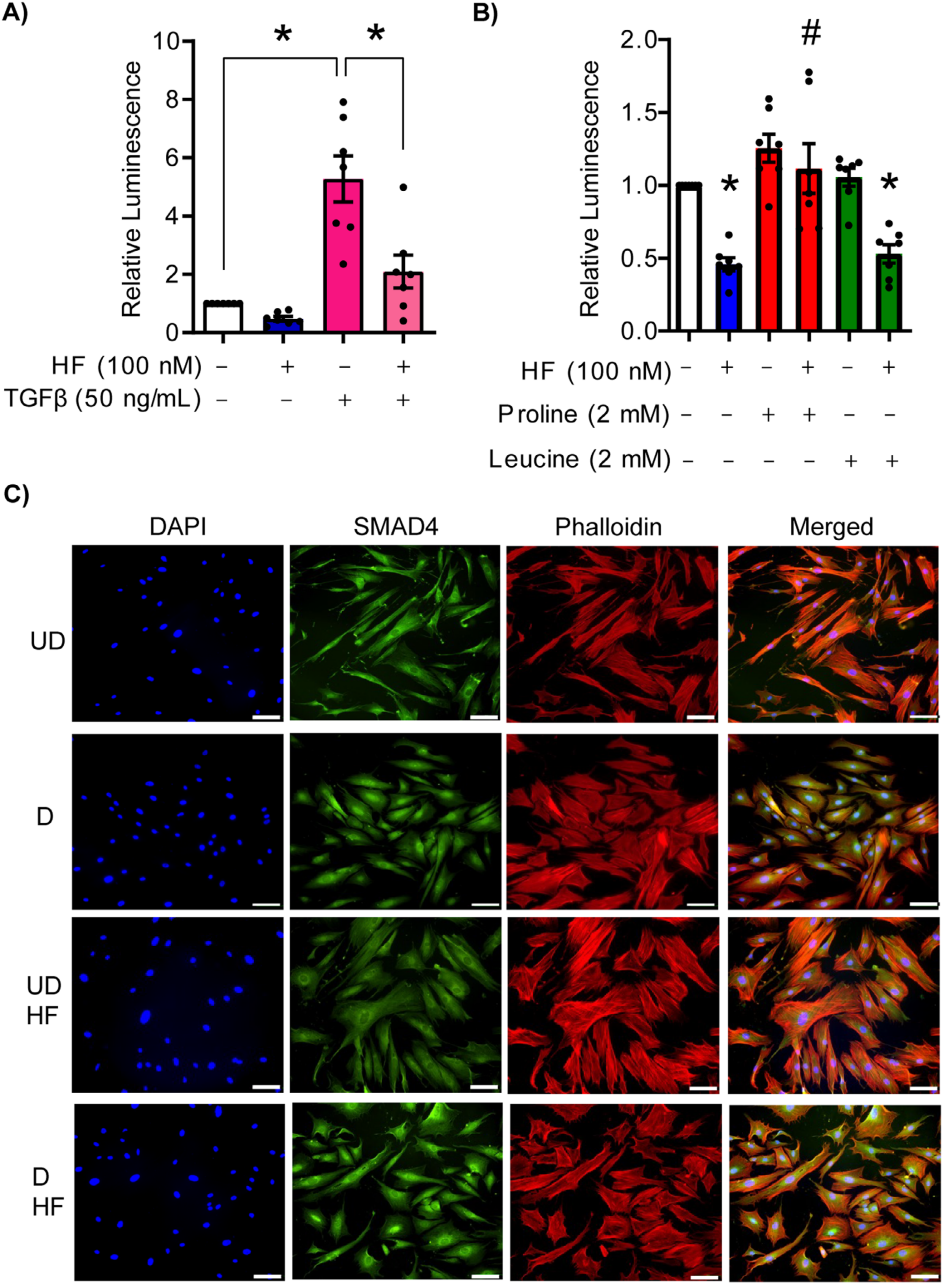
HF disrupts TGFβ‐SMAD signalling. (A) HEK293T cells were transfected with the SBE luminescence reporter [NlucP/SBE/Hygro] and then exposed to 50 ng/mL of TGFβ1, with or without 100 nM HF for 2 h. Luminescence was then measured. Please note that luminescence (and therefore SBE activation) was increased in cells treated with TGFβ1, and this was mitigated in cells treated with HF. (B) Cells transfected with the SBE luminescence reporter were exposed to HF and supplemented with proline or leucine, and then luminescence was measured. (C) Immunofluorescence for SMAD4 was performed in HESCs exposed to control media (undecidualized, UD) or decidualizing media (D), in the presence or absence of 100 nM HF. Cells were counterstained with phalloidin to detect actin and DAPI to detect nuclei. Scale bar = 100 μm. Data are presented as means ± SEM. Asterisks represent statistical significance versus untreated cells (*; *p* < 0.05), and number signs represent statistical significance when comparing HF‐treated cells to those supplemented with proline or leucine (#; *p* < 0.05), based on one‐way ANOVA with Tukey's multiple comparisons test. *N* = 7 independent replicates for panels (A) and (B), *N* = 3 independent replicates for panel (C). HESCs, human endometrial stromal cells; HF, halofuginone.

When TGFβ binds to its receptor, the activated TGFβ receptor phosphorylates SMAD2 and SMAD3, which then form a triplex with SMAD4. This complex translocates to the nucleus and acts as a transcription factor by binding upstream of target genes. To evaluate TGFβ‐SMAD signalling activation, we measured luminescence in cells transfected with a plasmid encoding nano‐luciferase downstream of a minimal promoter containing three copies of the SBE. HEK293T cells were used for these experiments because of their high transfection efficiency. To validate the specificity and reliability of the luciferase reporter in stimulating the TGFβ‐SMAD pathway, cells were treated with human recombinant TGFβ1, which significantly increased relative luminescence by more than fivefold compared to cells not receiving TGFβ1. HF inhibited this response, confirming that it effectively inhibits TGFβ‐SMAD signalling (*p* < 0.05; Figure 6A).

Next, we determined whether proline supplementation could rescue TGFβ‐SMAD signalling in the presence of HF without TGFβ stimulation. Compared to HF treatment alone, which reduced luminescence by approximately twofold (*p* < 0.05), proline supplementation restored luminescence, and therefore TGFβ‐SMAD signalling activity, to levels similar to controls (*p* < 0.05 compared to cells treated with HF). Leucine supplementation, on the other hand, did not have any effect (Figure 6B). Treating cells with proline or leucine in the absence of HF did not alter TGFβ‐SMAD signalling (Figure 6B). These results indicate that the TGFβ‐SMAD signalling pathway is sensitive to depleted proline availability and plays a role in modulating proline‐dependent processes.

### 3.8 SMAD4 Nuclear Localization Increases During Decidualization and Is Disrupted by HF

Using immunofluorescence, we assessed TGFβ‐SMAD signalling in HESCs by using SMAD4 nuclear translocation as an indicator of pathway activation. After 4 days of culture in decidualization medium, HESCs morphed from spindle‐like to ovoid shape marking successful decidualization, as shown using phalloidin staining of actin filaments. There was also an apparent increase in nuclear localization of SMAD4 in decidualizing HESCs, signifying activation of the TGFβ‐SMAD pathway (Figure 6C). In HESCs cultured in decidualization medium with HF, SMAD4 nuclear localization was reduced, with the protein showing distinct perinuclear and cytoplasmic localization. The reduced SMAD4 nuclear localization is consistent with the notion that HF inhibits TGFβ‐SMAD signalling, which may contribute, at least in part, to the disruption of HESC decidualization.

### 3.9 Gene Association Network Analysis Reveals Key Molecular Functions Modulated by HF in Decidualising HESCs

**FIGURE 7:**
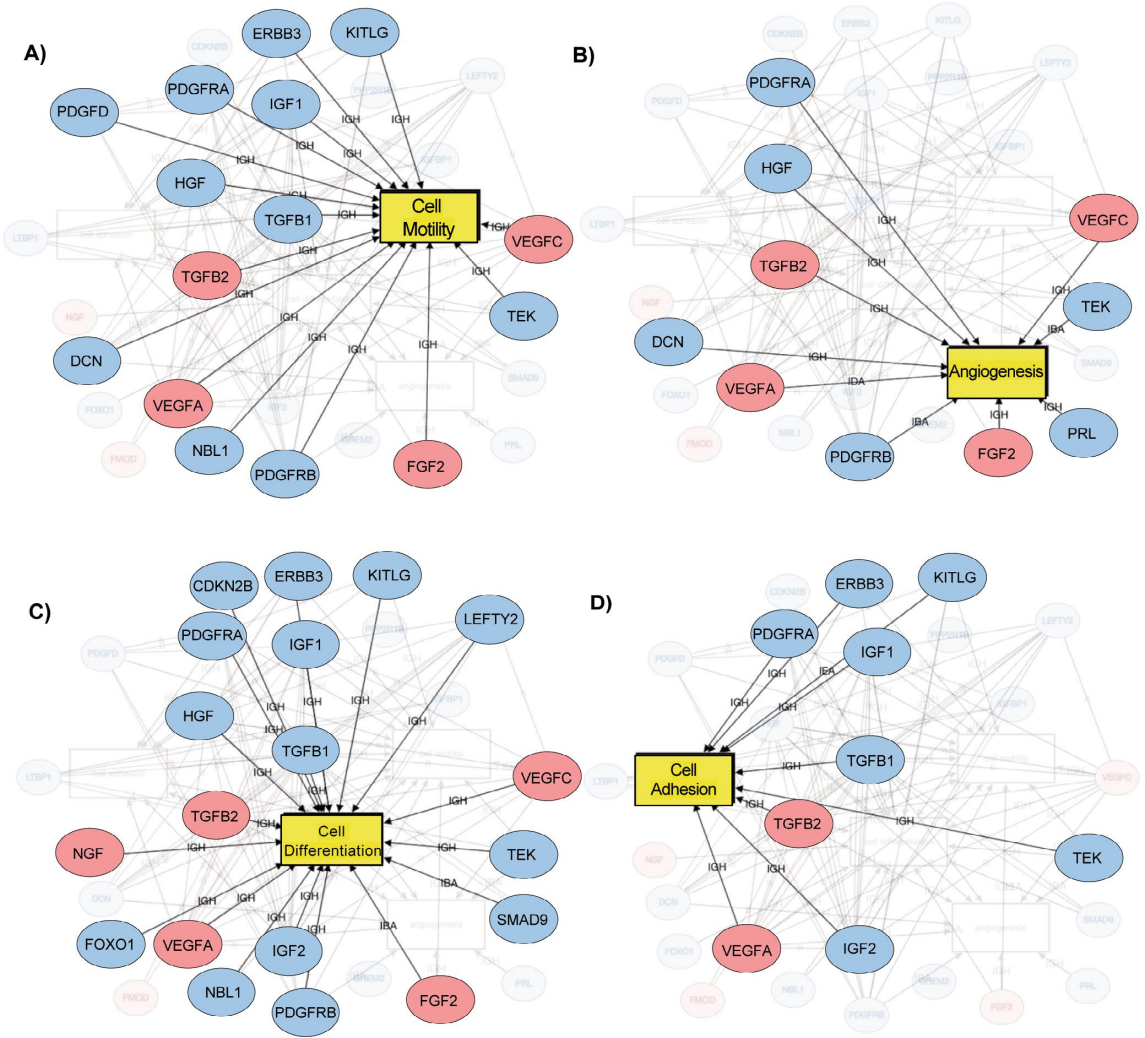
Network analysis of differentially expressed genes in decidualizing HESCs altered by HF. Network linking growth factors, receptor tyrosine kinases, markers of decidualization, and elements of the TGFβ signalling pathway based on genes that were differentially expressed in HESCs cultured in decidualization conditions in the presence or absence of HF. Genes mapping to proteins with molecular functions associated with (A) cell motility, (B) angiogenesis, (C) cell differentiation and (D) cell adhesion. Genes in red show increased expression in HF‐treated cells, whereas those in blue have decreased expression. HESCs, human endometrial stromal cells; HF, halofuginone.

Using iPathway Guide, we constructed an association network of genes encoding growth factors, receptor tyrosine kinases and decidualization markers that showed altered expression in HESCs cultured in decidualization medium with HF. The network predicted protein interactions and associated molecular functions (Table S3). As expected, differentiation was a key annotated function, with downregulation of several growth factors and TGFβ signalling members. Other annotated functions included motility, angiogenesis, and adhesion, all critical to HESC decidualization and function (Figure 7A–D).

## 4 Discussion

This study uncovered how HESC decidualization is affected by amino acid restriction, using HF to model reduced amino acid availability. HF inhibited decidualization, as evidenced by the lack of morphological changes into decidualized cells and reduced total and phosphorylated IGFBP‐1 secretion. Intriguingly, this inhibition of IGFBP‐1 may be cell‐type specific. HF stimulates IGFBP‐1 secretion in HepG2 cells, a commonly used model for fetal liver cells [24], through perturbation of stress pathways, such as mTOR or MAPK pathways, which are involved in IGFBP‐1 induction. These findings align with animal studies showing that isocaloric maternal protein or amino acid deprivation during pregnancy reduces birth weight [25, 26, 27] and amino acid levels in fetal plasma [28], which are associated with reduced IGF‐1 and increased IGFBP‐1 expression levels in the fetal liver [29]. Thus, perturbations of the IGF‐IGFBP‐1 signalling axis in both decidua and fetal liver may link amino acid restriction to obstetric complications.

Proline supplementation counteracted the inhibitory effect of HF on HESC decidualisation, whereas leucine supplementation did not. This highlights the specificity of HF in disrupting proline‐dependent processes and underscores the critical role of proline availability in driving HESC decidualisation. Proline enhances protein synthesis by activating the mTORC1 pathway [30], regulates oxidative stress [31], and is a key substrate for synthesis of polyamines in the placenta, which are essential for cell growth.

Metabolomic profiling has shown that proline levels in maternal blood during early pregnancy (5–14 weeks) are higher compared to non‐pregnant women [32], reaching a mean of 118 μmol/L during mid‐gestation [33]. During labour, significant enrichment of proline metabolism pathways is evident [34], suggesting that proline‐related processes may regulate key aspects of pregnancy from early stages through to parturition. Reduced proline levels are evident in women diagnosed with gestational diabetes and hypertension without proteinuria [35, 36]. Amniotic fluid levels of proline are lower in fetal growth restriction [37]. Some studies have also reported decreased proline in maternal serum [38] and urine [39] from women with preeclampsia, although other studies report the opposite [40, 41]. Nevertheless, proline appears to have an important role in pregnancy‐related processes, including its contribution to decidualisation as shown in the current study.

While there are no established guidelines presently for proline supplementation in human pregnancy, findings from pregnant animals provide supportive evidence for proline's potential benefits. In C57Bl/6 mice, dietary supplementation with 0.5% L‐proline (w/w with feed) enhances fetal survival, placental nutrient transport and reproductive outcomes [42]. In swine, supplementation with 0.89%–1.89% proline improves fetal weight, litter size and placental vascularisation, particularly in obese animals at higher risk of suboptimal pregnancy outcomes [43]. Short‐term proline supplementation around implantation has also been shown to improve birth outcomes in sows [44]. These findings suggest that proline supplementation within a range of 0.5%–1.89% of total dietary intake may be beneficial for pregnancy in mammals. While promising, further studies are needed to establish safe and effective dosages for clinical use.

HF canonically activates the amino acid starvation response through one of two pathways: induction of the integrated stress response through phosphorylated GCN2, or inhibition of TGFβ‐SMAD signalling. RNA sequencing analyses revealed upregulation of multiple TGFβ‐SMAD signalling components during decidualization, while expression of genes associated with the integrated stress response like *ATF4* and *EIF2AK4* was not significantly changed. TGFβ signalling is critical for decidualization of HESCs [45, 46, 47]. In mice, conditional ablation of *Tgfbr1* in the uterus impairs decidual immune cell function necessary for establishing maternal immune tolerance [48]. SMADs are key mediators of the TGFβ signalling response, but their precise role in decidualization is not fully understood. Nuclear localization of SMAD4 increased during decidualization and was disrupted by HF, suggesting that activation of the amino acid starvation response may inhibit TGFβ‐SMAD signalling required for decidualization.

In HEK293T cells, HF disrupted TGFβ‐SMAD signalling, which is consistent with several other studies using distinct cell types [24, 49, 50, 51], and this inhibition was restored by proline supplementation. This suggests that TGFβ‐mediated biological functions are sensitive to amino acid availability, consistent with growing evidence that TGFβ signalling regulates amino acid metabolism, bioavailability and biosynthesis [18]. TGFβ stimulation in non‐small cell lung cancer cells alters intracellular amino acid levels and metabolism [52]. TGFβ also induces glutamine catabolism by inducing the expression of glutamine transporter and glutaminase 1 [53]. In NIH3T3 fibroblasts, proline biosynthesis from glutamine is stimulated by TGFβ‐SMAD signalling and reduces the levels of mitochondrial reactive oxygen species [54]. In future studies, it would be interesting to investigate whether the metabolite profile of HESCs is altered during decidualisation and following HF exposure.

Network analysis revealed that HF altered biological functions including cell differentiation, adhesion, motility and angiogenesis. Specifically, HF prevented decidualization‐associated increases in *IGF1* and *IGF2* gene expression, supporting the notion that disruption of the IGF signalling axis may impair decidualization and related functions such as motility or adhesion. HF also reduced expression of *HGF*, which encodes a mitogenic growth factor [55], as well as *PDGFD*, *PDGFRA* and *PDGFRB*, which regulate fibrosis, complement activation [56], and late stage angiogenesis through vessel stabilization [57]. Conversely, HF increased the expression of *FGF‐2*, *VEGFC* and *VEGFA* [58, 59]. These data reveal how HF treatment affects the expression and signalling pathways of specific growth factors to alter important biological functions in HESCs.

The partial phenotypic overlap between HF treatment and leucine deprivation suggests that decidualisation may be sensitive to different amino acid deficiencies, with individual amino acids modulating this process through both shared and distinct pathways. Future studies are needed to dissect the specific signalling mechanisms and metabolic checkpoints involved in amino acid sensing during decidualisation, including whether HF modulates pathways such as mTOR, GCN2 or TGF‐β/SMAD signalling pathways in an amino acid‐specific or general manner.

While these data strengthen our understanding of the nutrient‐sensing pathways regulating decidualisation, certain limitations of this study should be acknowledged. First, we used an endometrial cell line, which may not entirely replicate in vivo decidualised HESCs [60]. While these cells allow for experimental consistency and long‐term passaging, and we have verified their decidualisation capacity, it would be informative to repeat some of our experiments in primary HESCs isolated from endometrial biopsies. Second, HF treatment may not fully recapitulate the biological effects of amino acid restriction on decidualisation. While HF is a well‐established tool to mimic amino acid (particularly proline) starvation, validating our findings in physiologically relevant in vivo contexts remains an important future direction. Although administering HF to pregnant animals may introduce confounding systemic effects, future studies could investigate the role of TGF‐β/SMAD signalling in decidualisation deficiencies using an animal model of protein or nutrient deficiency.

In conclusion, our findings suggest that amino acid restriction activates pathways in HESCs that interfere with their capacity to undergo decidualisation. These findings provide crucial insights into the relationship between nutrient stress and pregnancy complications associated with defective decidualisation. Our results underscore the significance of amino acids such as proline and specific growth factors in driving decidualisation and help to inform the intricate molecular mechanisms governing decidualisation in HESCs under nutrient‐stress conditions.

## Author Contributions

**Chloe Jang:** data curation (lead), formal analysis (lead), investigation (lead), methodology (lead), project administration (lead), validation (lead), visualization (lead), writing – original draft (lead), writing – review and editing (lead). **Cristiana Iosef:** data curation (supporting), formal analysis (equal), methodology (supporting), project administration (supporting), visualization (supporting), writing – review and editing (supporting). **Stephen J. Renaud:** conceptualization (equal), investigation (supporting), methodology (supporting), project administration (equal), supervision (supporting), writing – review and editing (equal). **Victor K. M. Han:** conceptualization (equal), funding acquisition (lead), investigation (lead), project administration (lead), resources (equal), supervision (lead), validation (lead), visualization (lead), writing – review and editing (equal).

## Conflicts of Interest

The authors declare no conflicts of interest.

## Supporting information


**Data S1:** jcmm70821‐sup‐0001‐Supinfo.docx.

## Data Availability

The raw and processed RNA sequencing data from this study can be accessed in the Gene Expression Omnibus (GEO) at GSE294481.
