# Supplementary material for: Amino Acid Restriction Impairs Human Endometrial Stromal Cell Decidualization and Is Rescued by Proline Supplementation
Source: J Cell Mol Med. 2025 Sep 16;29(18):e70821. doi: 10.1111/jcmm.70821 (PMC12439679; doi:10.1111/jcmm.70821)
Supplement: Supplementary file 1 — Data S1: jcmm70821‐sup‐0001‐Supinfo.docx. [file JCMM-29-e70821-s001.docx]

**Supplemental Figures:**

**Figure 1. Effect HF and proline or leucine on cellular integrity.** HESC were cultured in decidualization medium and treated with HF with or without proline or leucine supplementation, and then the number of viable cells was estimated using a MTT assay. Data represent mean ± SEM, n = 3.

**
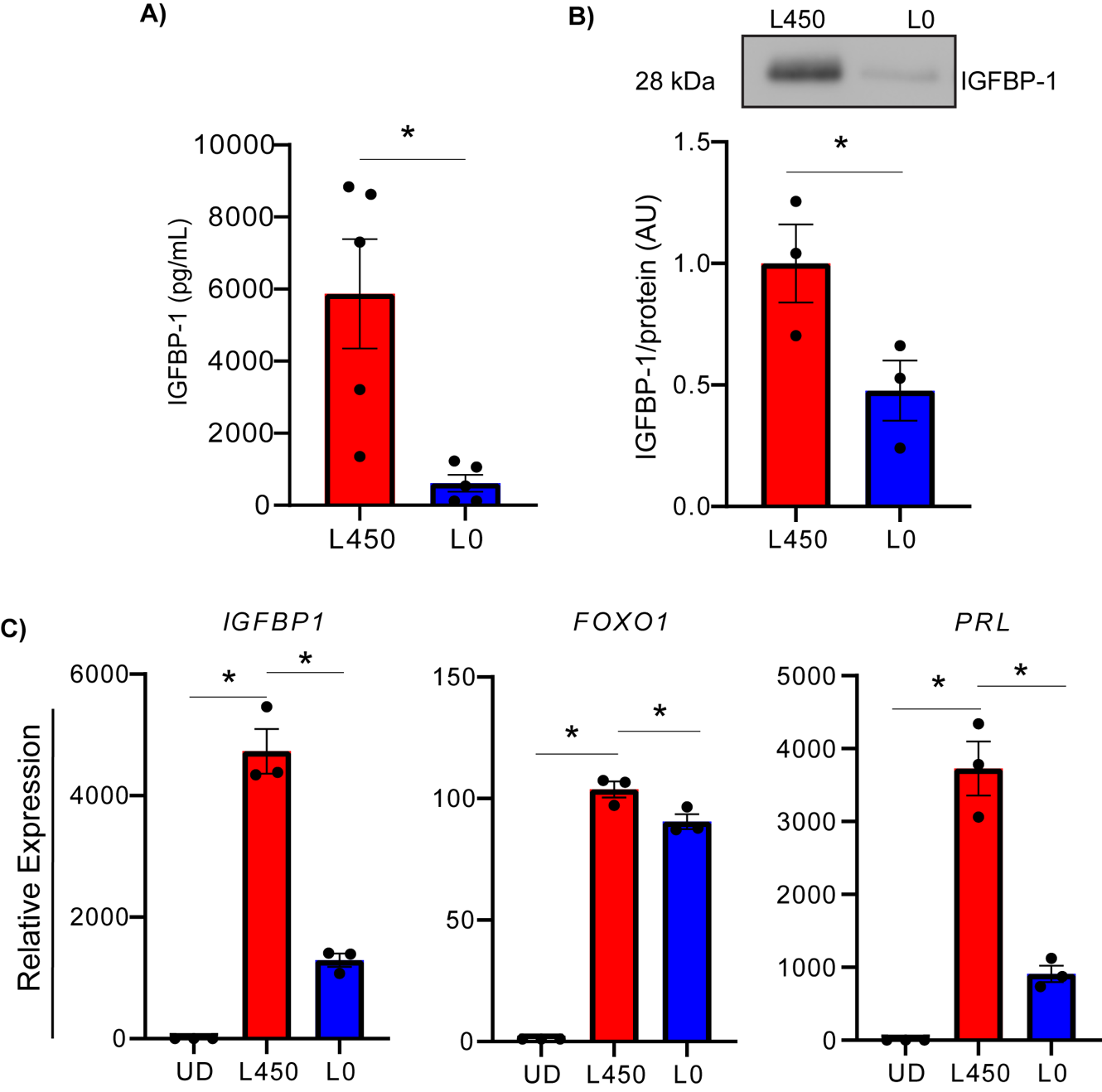
**

**Figure 2. Leucine deprivation impairs decidualization.** HESCs were cultured for 6 days in leucine-replete (450 μM) or leucine-depleted (0 μM) decidualization media. A. IGFBP-1 levels in conditioned media. B. Western blot analysis of cellular IGFBP-1 protein levels. C. Transcript levels of IGFBP1, PRL, and FOXO1 in HESCs cultured in leucine-replete or leucine-depleted decidualization media. Undecidualized (UD) cells cultured in leucine-replete media are also shown. Data represent mean ± SEM. N=5 independent replicates for panel A, N=3 independent replicates for panels B-C. *p < 0.05.


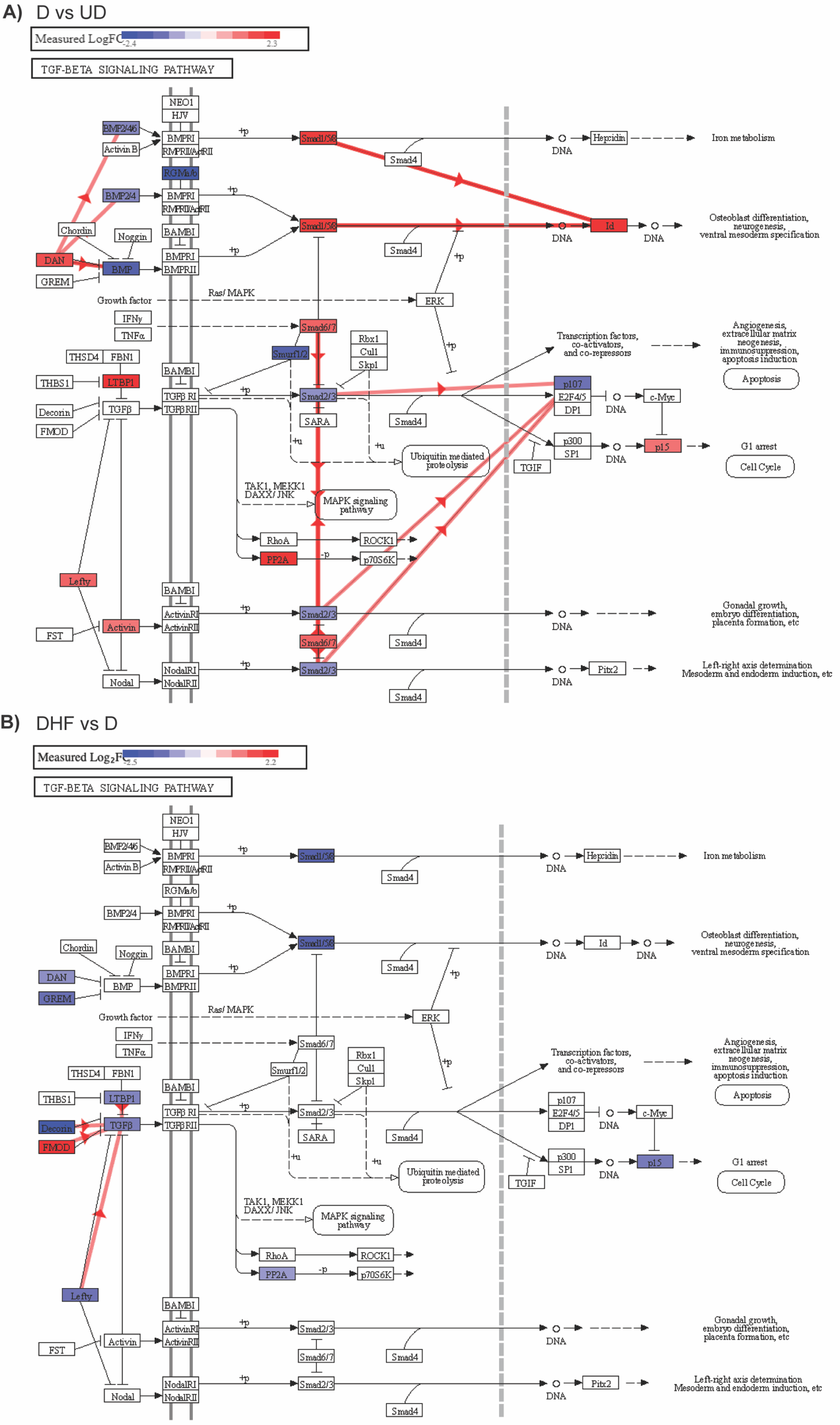


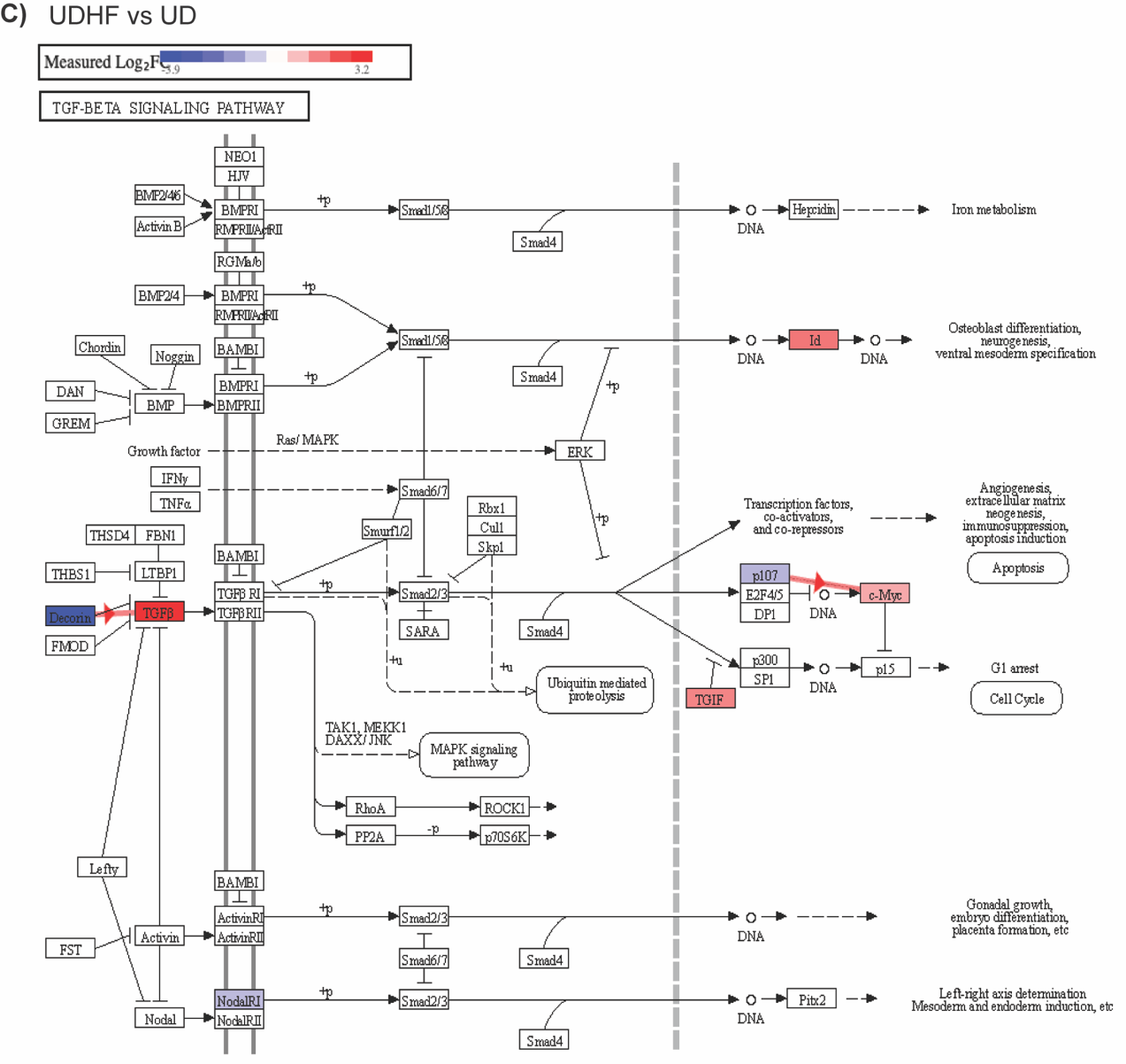


**Figure 3. Pathways analysis of the effect of HF on TGFβ signaling.** IPathway Guide analysis revealed measured Log2FC of key signaling members of the TGFβ pathway as well the predicted downstream cascade effects for A. Decidualizing HESCs vs Undecidualized HESCS (**D** vs **UD**), B. D treated with HF vs D (**D HF vs D),** and C. UD treated with HF vs UD (**UD HF vs UD).**

**Supplemental Table 1. List of Antibodies Used**

| **Antibody List** | | |
| --- | --- | --- |
| **Primary Antibody** | **Catalogue Number (Dilution)** | **Manufacturer** |
| IGFBP-1 6303 SP-5 | 100056 (mouse mAb); Immunocytochemistry (ICC) 1:200; Western blotting 1:10,000 | Medix Biochemica, Espoo, Finland |
| Phospho-IGFBP-1 (Ser101) | Custom antibody (Western blotting 1:1000) | YenZym Antibodies, Brisbane, CA, USA |
| Vimentin | ab92547 (rabbit pAb); ICC 1:500 | Abcam, Cambridge, UK |
| SMAD4 | 46535S (rabbit mAb); ICC 1:500 | Cell Signaling Technology, Danvers, MA, USA |
| **Secondary Antibody** | **Catalogue Number** | **Manufacturer** |
| Donkey anti-Mouse IgG | Alexa Fluor 594; R37115 (2 drops/mL) | Thermo Fisher Scientific, Waltham, MA, USA |
| Donkey anti-Rabbit IgG | Alexa Fluor 488; R37118 (2 drops/mL) | Thermo Fisher Scientific, Waltham, MA, USA |

**Supplemental Table 2. List of Primers Used**

| **Primer List** |  |  |
| --- | --- | --- |
| **Gene** | **Forward Primer (5'→3')** | **Reverse Primer (5'→3')** |
| ATF4 | CATGGCGTATTAGGGGCAGC | GCTGCTGAATGCCGTGAGAA |
| B-ACTIN | GAGCACAGAGCCTCGCCTTT | GCGCGGCGATATCATCATCC |
| FOXO1 | CGAGTGGATGGTCAAGAGCGT | TTGCCACCCTCTGGATTGAGC |
| HAND2 | CACCAGCTACATCGCCTACC | ATTTCGTTCAGCTCCTTCTTCC |
| IGFBP-1 | CATCCTTTGGGACGCCATC | GGATGTCTCACACTGTCTGC |
| PRL | GGGGTTCATTACCAAGGCCA | TGGATAGGATAGCCTCCGGG |
| YWHAZ | ATGCAACCAACACATCCTATC | GCATTATTAGCGTGCTGTCTT |

**Supplemental Table 3. Association Network of Key DEGs Affected by HF in Decidualizing HESCs.**

| Function | Genes |
| --- | --- |
| Motility | Upregulated: *FGF2, TGFB2, VEGFA, VEGFC* |
|  | Downregulated: *DCN, ERBB3, HGF, IGF1, KITLG, NBL1, PDGFD, PDGFRA, PDGFRB, TEK, TGFB1* |
| Angiogenesis | Upregulated: *FGF2, TGFB2, VEGFA, VEGFC* |
|  | Downregulated: *DCN, HGF, PDGFRA, PDGFRB, PRL, TEK* |
| Differentiation | Upregulated: *FGF2, NGF, TGFB2, VEGFA, VEGFC* |
|  | Downregulated: *CDKN2B, ERBB3, FOXO1, HGF, IGF1, IGF2, KITLG, LEFTY2, NBL1, PDGFRA, PDGFRB, SMAD9, TEK, TGFB1* |
| Adhesion | Upregulated: *TGFB2, VEGFA* |
|  | Downregulated: *ERBB3, IGF1, IGF2,* *KITLG, PDGFRA, TEK, TGFB1* |
